# Combined hip arthroscopy with periacetabular osteotomy for hip dysplasia: a systematic review

**DOI:** 10.1093/jhps/hnae016

**Published:** 2024-04-18

**Authors:** Kenneth J Lukas, Reza Ojaghi, Kednapa Thavorn, Sasha Carsen, Kevin Smit, Paul E Beaulé

**Affiliations:** Division of Orthopaedics Surgery, The Ottawa General Hospital, 501 Smyth Rd, Ottawa, ON K1H 8L6, Canada; Division of Orthopaedics Surgery, The Ottawa General Hospital, 501 Smyth Rd, Ottawa, ON K1H 8L6, Canada; The Ottawa Hospital Research Institute, 1053 Carling Ave, Ottawa, ON K1Y 4E9, Canada; The Children’s Hospital of Eastern Ontario, 401 Smyth Rd, Ottawa, ON K1H 8L1, Canada; The Children’s Hospital of Eastern Ontario, 401 Smyth Rd, Ottawa, ON K1H 8L1, Canada; Division of Orthopaedics Surgery, The Ottawa General Hospital, 501 Smyth Rd, Ottawa, ON K1H 8L6, Canada

## Abstract

Periacetabular osteotomy (PAO) is a surgical procedure that corrects acetabular dysplasia without necessarily addressing intra-articular pathology. Hip arthroscopy is being increasingly used to address soft tissue pathologies at the time of a PAO. This review aims to determine patient-reported outcome measure scores (PROMs) of combining hip arthroscopy and PAO. This systematic review followed the Preferred Reporting Items for Systematic Review and Meta-Analyses guidelines to identify English studies that reported upon patient populations that had PAO’s performed with arthroscopy at the time of surgery for correcting developmental hip dysplasia. We identified 428 articles; 14 full-text articles met the inclusion criteria. Between 2011 and 2022, 1083 hips from the selected articles underwent a combined PAO and arthroscopic procedure, with a mean follow-up of 3.7 years. Of the studies that reported it, 63% of the evaluated population were found to have labral tears that required either labral repair (49%), labral debridement (12%) or combined procedure. Multiple PROMs were identified in the literature, with no standardized reporting system used between articles. All articles reported statistically improved patient-reported outcomes from a combined PAO and arthroscopy procedure. There was no difference in PROMs when comparing PAO performed with or without arthroscopy. One study suggested superior outcomes for active individuals who underwent PAO and arthroscopy. Patient-reported outcome scores improve significantly after PAO with or without arthroscopy, with no differences in adverse events, and only limited evidence that active individuals benefit from labral repair.

## INTRODUCTION

The abnormal morphology of hip dysplasia presents with reduced coverage of the femoral head that can lead to overloading of the rim cartilage and labrum-creating-associated instability [1, 2]. The natural history of this chronic mechanical overload includes the development of labral damage, cartilage wear, progressive hip pain and eventual progression to degenerative arthritis causing significant disability and pain [1–4].

Periacetabular osteotomy (PAO) is a well-described surgical technique that enables correction of the bony acetabular deficiency seen in hip dysplasia. This is performed by reorientating the native acetabulum to improve the femoral head coverage to create an improved mechanical appropriate position that decreases shear forces and loads along the acetabular rim [1, 5–7]. The PAO, regardless of surgical approach, offers an excellent joint-preserving treatment option for young, pre-arthritic, symptomatic patients with acetabular dysplasia, with good-to-excellent clinical and radiographical results post-intervention in acute, mid-term and long-term follow-up [1, 2, 8–10].

The high incidence of intra-articular pathology occurring in the presence of hip dysplasia during PAO was reported by Klaue *et al*. and referred to as acetabular rim syndrome [11]. The authors described elevated shear forces across the labrum and cartilage due to increased abnormal edge loading that predisposes the hip joint to developing intra-articular pathologies [3, 6, 11]. In the literature, the incidence of symptomatic labral tears in dysplastic hips treated with PAO that were evaluated through open arthrotomy or arthroscopy ranges from 60% to 98% of patients undergoing a PAO [3, 5–7]. Although a large proportion of patients have excellent outcomes with PAO alone, there remains a subset who continue to report unfavourable outcomes which is believed to be secondary to unaddressed intra-articular soft tissue pathologies that require the need for repeat surgical intervention [12, 13].

Hip arthroscopy allows for a minimally invasive approach to diagnose and address intra-articular pathologies of the cartilage, labrum, femoral head and acetabulum that may not be corrected by PAO alone [2, 3, 8]. Compared to open arthrotomy, hip arthroscopy offers improved visualization of intra-articular pathology with less dissection and quicker recovery [8]. In addition, hip arthroscopy allows for the correction of femoroacetabular impingement morphology which can be co-existent with dysplasia, and correction of abnormal femoral head/neck bony deformity and associated soft tissue pathologies that can be addressed at the same setting [7]. However, it remains controversial whether repair of cartilage pathologies should be done at the time of PAO, due to reports of patients with labral tears being asymptomatic after undergoing PAO alone [1]. Traditional PAO can also be paired with an open arthrotomy to address labral repairs, and has well-documented long-term successful outcomes [8], with the Wyles *et al*. and Panos *et al*. groups both reported that there are no difference in outcomes between an open versus arthroscopic procedures [14, 15]. Alternative studies have data showing that concomitant hip arthroscopy at time of PAO’s have favourable outcomes at short- and mid-term follow-up with no additional complications to PAO’s alone [12]. Furthermore, multiple studies have reported successful long-term outcomes of PAO procedures performed with no concomitant cartilage intervention, with researchers hypothesizing that the reorientation of the acetabular through an osteotomy facilitates biosynthetic environment through normalization for mechanical loading that leads to cartilage adaptation and chondrocyte regeneration [8].

The aim of this systematic review is to summarize the existing evidence on clinical and patient-reported outcomes in patient cohorts that have undergone hip arthroscopy as adjunct to a PAO to establish if this combined procedure creates superior reported outcomes compared to PAO alone.

## METHODS

### Search strategy

This systematic review protocol followed the Preferred Reporting Items for Systematic Review and Meta-Analyses (PRISMA) guidelines. The following databases were searched: MEDLINE and PUBMED. Search terms were entered into three concepts: concept 1 included terms: ‘hip dysplasia’, ‘hip dislocation’, ‘hip’, ‘dysplasia’. Concept 2 included terms: ‘periacetabular osteotomy’, ‘periacetabular’, ‘osteotom’, ‘osteotomied’, ‘osteotomy’. Concept 3 included terms: ‘systematic review’, ‘scoping review’ and ‘meta-analysis’. In addition, filters were applied which included: ‘Adult: 18+ years’. Terms within each concept were combined using OR Boolean operator and the three concepts were combined with the AND Boolean operator. Terms were searched using title and abstracts.

### Study eligibility criteria

Articles were included based on the following criteria: (i) English language, (ii) commented on parameters or diagnostic testing of hip dysplasia, (iii) performed both hip arthroscopy and PAO during the same surgical procedure, (iv) contained outcome data on patients undergoing the surgical procedure, (v) had a sample size of at least 10 patients and (vi) were published since 2000.

Articles were excluded if they involved any of the following: (i) review articles, technique articles, (ii) articles with overlapping patient populations (e.g. same cohorts used for multiple studies), (iii) articles including patients with a mean age <18 years, (iv) patients with any concomitant hip conditions (e.g. Legg–Calve–Perthes, slipped capital femoral epiphysis, or post-infectious hip deformities), (v) patients with genetic or neuromuscular causes of hip dysplasia, (vi) patients with Tonnis grade 2 or higher arthritic changes in the hips, (vii) articles covering femoral osteotomy and (viii) articles on previous surgical intervention as an adult or child.

All articles retrieved by database searches were uploaded and duplicated articles were removed using an online systematic review tool (Covidence, Melbourne, Australia). Articles were then appraised against the inclusion and exclusion criteria using a two-step technique. Two reviewers independently reviewed the titles and abstracts to select relevant articles for full-text review. Articles without abstracts were chosen for full-text review by default. Both reviewers then examined the full-text articles for eligibility.

### Data extraction

Data from included studies were extracted by a single reviewer and verified by a second reviewer. Data collected from the articles included: patient demographics, mean follow-up period, radiographic measurements, patient-reported outcome scores and complications. We defined patients with dysplasia as having a lateral center edge angle (LCEA) <25 and in conjunction with the Ottawa Classification for symptomatic acetabular dysplasia [16].

### Data analysis

We compiled a table to aid in the evidence synthesis and evaluate the study heterogeneity of the extracted data for each included study. Given the heterogeneity in patient characteristics, study types and outcome definitions, a primary narrative synthesis was performed.

## RESULTS

**Fig. 1.**
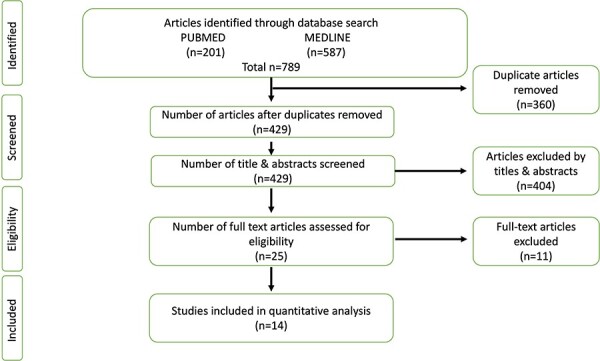
PRISMA flow diagram for articles that assessed PAO & Arthroscopy

The search of the electronic databases identified 789 articles; of which there were 360 duplicates, leaving 429 articles for review. From these articles, 404 were excluded based on titles and abstracts, leaving 25 articles for full-text review. Eleven articles were excluded (6 for non-arthroscopic procedures, 4 wrong study design and 1 non-English), leaving 14 articles [2–4, 6–8, 12, 17–23] that fit the inclusion criteria and were appropriate for data extraction (Fig. 1).

The 14 articles [2–4, 6–8, 12, 17–23] included for the review were published between 2011 and 2023, and were identified as level III or IV evidence (Table I), with no obvious duplications of cohorts. Within the 14 articles combined there was a total of 1055 patients who underwent PAO’s with arthroscopy, with a total of 1083 hips, of which 28 patients proceeded with staged bilateral PAO’s. The mean age of all participants was 28.4 ± 8.5 (range: 12–67). The preoperative mean LCEA and alpha angle were 14.3 ± 6.0 (range: 9–26) and 55.9 ± 11.2 (range: 32–90), respectively. The accumulative mean average follow-up was 3.8 years (range: 0.6–18) (Table IV).

**Table I.** Studies demographics and pre-operative radiographic measurements

*Study*	*Year*	*Level of evidence*	*Patients (n)*	*Hips (n)*	*Mean Age (years)*	*SD*	*Range (years)*	*Mean LCEA*	*SD*	*Range*	*Mean Alpha*	*SD*	*Range*
Fuji *et al*.	2011	IV	121	121	40	X	13–64	−0.7	8.5	X	X	X	X
Kim *et al*.	2011	III	40	43	40	X	20–67	7.3	X	10–19	X	X	X
Domb *et al*.	2014	IV	16	16	21	6.7	12–33	11	7.5	10–17	X	X	X
Domb *et al*.	2015	IV	17	17	24	7.1	X	29.1	4.2	X	X	X	X
Ricciardi *et al*.	2016	III	21	24	27	X	12–41	18	X	15–21	54	X	44–62
Goronzy *et al*.	2017	III	74	85	X	X	X	16.3	5.8	5–21	56	15	33–90
Hellman *et al*.	2018	III	56	56	30	9.8	X	X	X	X	X	X	X
Maldonado *et al*.	2019	IV	16	16	24	6.8	12–35	14.2	6.7	X	56	12	X
Sabbag *et al*.	2019	IV	240	248	27	9.2	X	18.3	2	X	X	X	X
Wasko *et al*.	2019	IV	275	275	24	X	X	17.7	X	15–20	X	X	X
Cho *et al*.	2020	IV	36	39	37	11.3	16–59	8.7	X	9–18	X	X	X
Shamrock *et al*.	2021	IV	61	61	24	8.5	X	X	X	X	X	X	X
Jimenez *et al*.	2022	IV	29	29	26	4.7	X	16.5	6.3	X	56	10	X
Fleming *et al*.	2023	III	53	53	27	X	13–47	16.8	6.9	X	58	8	X

X, not reported.

Nine [2–4, 6–8, 17, 18, 21] of the included papers commented on the intra-operative condition of the labrum, identifying 63% (392/617) of all the participants having labral tears. These were treated with labral repair in 49% (308/617) and labral debridement in 12% (74/617) [2–4, 6–8, 17, 18, 21].

Seven [2, 3, 6, 8, 12, 20, 22] studies reported on intra-operative femoral and acetabular cartilage damage. These studies included a total of 536 participants, of which 29% (158/536) had femoral head cartilage damage confirmed intra-operatively. Seven studies reported intra-operative acetabular cartilage damage cumulatively that included a total of 553 participants, of which 79% (437/552) were reported to have cartilage damage present. Debridement accounted for a majority of management of the femoral and acetabular lesions at 63% of the reported participants (98/156) [2, 6, 7, 17, 18] and microfracture was the reported treatment in 13% (12/89) of the reported study populations. Two studies [20, 21] reported on performing femoral osteoplasty in 70% (185/264) of their participants. Four studies [7, 18, 20, 21] reported on performed acetabular osteoplasty in their patient populations, accounting for 35% of these studies total participants (Table II).

**Table II.** Intra-operative findings and management options for labral and chondral lesions

*Study*	*PAO (n = )*	*Femoral osteoplasty (n)*	*Acetabular osteoplasty (n)*	*Labrum pathology (n)*	*Labrum repaired (n)*	*Labrum debrided (n)*	*Femoral cartilage damage (n)*	*Acetabular cartilage damage (n)*	*Cartilage debrided (n)*	*Cartilage microfracturing (n)*
Fuji *et al*.	121	X	X	54	54	0	34	71	X	X
Kim *et al*.	43	X	X	54	16	38	X	X	38	X
Domb *et al*.	16	X	X	25	6	9	14	2	9	X
Domb *et al*.	17	X	3	17	12	5	X	X	5	3
Ricciardi *et al*.	24	X	9	13	0	13	X	X	23	X
Hellman *et al*.	56	X	16	25	16	9	7	28	18	6
Maldonado *et al*.	16	10	X	X	X	X	16	16	15	3
Sabbag *et al*.	248	175	95	150	150	0	X	X	X	X
Wasko *et al*.	275	X	X	X	X	X	52	253	X	X
Cho *et al*.	39	X	X	1	1	0	39	39	X	X
Jimenez *et al*.	29	X	X	X	X	X	3	28	X	X
Fleming *et al*.	53	X	X	53	53	X	X	X	X	X

X,  not reported.

Several articles reported on patient-reported outcome measure scores (PROMs), of which, all identified statistically significant improvements from pre- to post-operative intervention (Tables III and IV). Variable PROMs were used that included: Hip Disability and Osteoarthritis Outcome scores (HOOS) symptoms, Hip Disability and Osteoarthritis Outcome scores Activities of Daily Living (HOOS ADL), Hip Outcome Scoring Sport Scale (HOS-SSS), Western Ontario and McMaster Universities Osteoarthritis Index (WOMAC), Visual Analogue Scale (VAS), International Hip Outcome Tool-12 or 33 (iHOT-12/33), Modified Harris Hip Score (mHHS) and Non Arthritic Hip Score (NAHS) [2–4, 6–8, 12, 17–23].

**Table III.** Pre-operative patient-reported outcome scores

*Study*	*Patient (n)*	*Hips (n)*	*HOOS symptoms*	*HOOS pain*	*HOOS ADL*	*HOOS S&R*	*HOS-SSS*	*HOOS QoL*	*WOMAC*	*VAS*	*iHOT 12/33*	*mHHS*	*NAHS*	*HOS-SSS 2*
Kim *et al*.	40	43	X	X	X	X	X	X	X	X	X	72.4	X	X
Domb *et al*.	17	17	X	X	65.4	X	37.7	X	X	5.6	X	63.9	57.7	37.7
Ricciardi *et al*.	21	24	X	X	X	X	41	X	X	X	30	58	X	41
Maldonado *et al*.	16	16	X	X	X	X	37.6	X	X	5.8	X	63.5	56.8	X
Wasko *et al*.	275	275	50	47.5	63.2	37.5	X	25	61.5	X	X	61.1	X	X
Cho *et al*.	36	39	X	X	X	X	X	X	X	X	X	72	X	X
Jimenez *et al*.	29	29	X	X	X	X	X	X	X	5.1	X	61.3	61.2	43.3
Fleming *et al*.	53	53	X	X	X	X	X	X	X	X	34.3	57.2	X	X

SSS, Sports-Specific Subscale; X, not reported.

The highest reported complication was lateral femoral cutaneous nerve dysaesthesia, which when stratified across all participants represented 1.2% (13/1083) of all cases (Table V). Infection represented the second highest complication at 0.9% (10/1083), followed by hypertrophic ossification at 0.7% (7/1083) and deep vein thrombosis/pulmonary embolism at 0.6% (6/1083). One participant (0.1%, 1/1083) required conversion of their PAO to a total hip arthroplasty (THA) due to continued hip pain for 8 years after their POA [8].

**Table IV.** -operative patient-reported outcome scores and mean follow-up durations

*Study*	*Patient (n)*	*Hips (n)*	*Mean Follow-up (years)*	*HOOS symptoms*	*HOOS ADL*	*HOS-SSS*	*WOMAC*	*VAS*	*IHOT-12/33*	*mHHS*	*NAHS*
Fuji *et al*.	121	121	9.9	X	X	X	X	X	X	X	X
Kim *et al*.	40	43	6	X	X	X	X	X	X	90	X
Domb *et al*.	16	16	1.5	X	X	X	X	X	X	X	X
Domb *et al*.	17	17	2.4	X	80.1	74.4	X	2.6	X	84	79.5
Ricciardi *et al*.	21	24	1.9	X	X	80	X	X	84	83	X
Goronzy *et al*.	74	85	5.3	X	X	X	90.6	X	X	X	X
Hellman *et al*.	56	56	3	31.8	X	X	X	X	X	X	X
Maldonado *et al*.	16	16	5.5	X	X	68.1	X	3.1	66.3	81.6	79.8
Sabbag *et al*.	240	248	3	X	X	X	X	X	X	X	X
Wasko *et al*.	275	275	3	X	X	X	X	X	X	X	X
Cho *et al*.	36	39	3.3	X	X	X	X	X	X	90	X
Shamrock *et al*.	61	61	X	X	X	X	X	X	X	X	X
Jimenez *et al*.	29	29	2.4	X	X	80.2	X	1.9	X	88.3	90
Fleming *et al*.	53	53	2.1	X	X	X	X	X	63.7	81.9	X

SSS, Sports-Specific Subscale; X, not reported.

**Table V.** Table of listed complications from studies

*Study*	*LFCN dysaesthesia (n)*	*Hyper-trophic ossification (n)*	*Sacroiliitis (n)*	*Infection (n)*	*Hematoma (n)*	*Hyper-trophic ossification requiring excision (n = )*	*DVT/PE (n)*	*Revision surgery (n)*	*PAO failure (n)*	*Conversion to THA (n = )*
Kim *et al*.	X	2	X	X	X	X	1	X	X	X
Domb *et al*.	X	X	1	2	X	X	1	X	X	X
Ricciardi *et al*.	X	1	X	X	X	X	X	X	X	X
Maldonado *et al*.	X	X	1	4	X	X	1	X	X	X
Sabbag *et al*.	13	3	X	3	1	1	1	X	X	X
Cho *et al*.	X	1	X	X	X	X	1	X	X	1
Shamrock *et al*.	X	X	X	1	X	X	1	X	X	X
Fleming *et al*.	X	2	X	X	X	X	X	1	X	X

LFCN, lateral femoral cutaneous nerve; DVT, deep vein thrombosis; PE,  Pulmonary embolism; THA ,  total hip arthroplasty; X, not reported.

## DISCUSSION

Concomitant arthroscopy during PAO has become far more common and generally accepted, and this systematic review highlights that a number of studies have investigated some of the risks and benefits of proceeding with a single comprehensive surgery including both procedures. Beaulé *et al*. reported on the impact of intra-articular pathology on PROMs in mid-term follow-up of PAO, where patients with a larger pre-operative alpha had lower scores post-operatively [24]. Ross *et al*. described the benefit of direct visualization of intra-articular pathology with arthroscopy, identifying labral tears in 65.8% and chondral lesions in 68.5% of their study population prior to performing directional osteotomy for dysplasia [6, 25]. Multiple other studies have echoed this advantage, and the ability to have improved diagnosis and treatment of peripheral and central compartment pathologies (e.g. chondral or labral lesions), and ability to perform arthroscopic head–neck femoral osteochondroplasty [2, 7, 8, 18]. The ability to visualize, diagnose and correct possible intra-operative pathology is thought to increase post-operative patient satisfaction and likelihood of long-term success of the PAO procedure [21]. However, some studies have neglected to correct intra-articular pathologies, choosing to solely perform PAO’s, and have reported successful long-term outcomes from PROMs [8–10]. In 2016, Riccardi *et al*. compared 22 hips that underwent a combined PAO and arthroscopy procedure to 56 hips that had a PAO performed alone. Their reported short-term follow-up found that there was no difference in patient PROMs, leading them to not recommend concomitant arthroscopy for labral repair in patient’s undergoing PAO [2, 18]. A similar paper published in 2018 by Hellman *et al*., also determined there was no difference in outcomes of individuals who had a chondral defect compared to those without one after undergoing a combined PAO and arthroscopic procedure [2]. This result may be explained by the critical importance of an adequate reorientation of the weight-bearing surface in hip dysplasia improving the biomechanics by redistributing mechanical stress over a larger contact area, providing a better cartilage environment for healing and durability [8]. A single-center retrospective review analyzed PROMs of patients who had hip arthroscopy performed after a PAO and found no significant improvement in their population, with 27% requiring conversion to a total hip arthroplasty (THA) [13, 26]. An additional study published by Cvetanovich *et al*. identified that patients with recurrent symptoms following a PAO who underwent an arthroscopic procedure for intra-articular pathology had reported improved hip pain and decreased stiffness, however, had no significant improvements on the outcome scores [27]. Overall, PAO correction of acetabular dysplasia has significant favourable PROMs results, though additional intra-articular pathology treatment with arthroscopy appears to lack clear evidence of support.

Concerns that arise when combining PAOs and arthroscopic procedures include: fluid extravasation of the joint tissue which may lead to more difficult surgical dissection, sciatic neuropraxia and double hit phenomenon with use of a traction table, a surgical learning curve for performing these surgeries, or the requirement of designated surgeon for each procedure, prolonged surgical times and associated risks (e.g. blood loss and infection), and increased risk of capsular adhesions [2, 7, 8, 18] as well as the overall increased cost. It is also generally agreed that arthroscopy alone to address symptoms of hip dysplasia is mainly reserved as a temporizing measure due to high rates of reoperation [2]. More importantly, little consideration has thus far been given to the additional direct and indirect additional costs of concomitant hip arthroscopy, which includes prolonged operative time as well as material costs.

The degenerative process in hip dysplasia long before the development of osteoarthritis (OA) becomes apparent on plain radiographs, and tends to originate on the acetabular side in the weight-bearing area of the labrum in the anterior–superior location between 2 o’clock and 3 o’clock [3, 17, 18]. Labral lesions are frequently present in patients with hip dysplasia, and articles report the association with worse outcomes if left untreated due to mechanical symptoms of catching and locking that are associated with the labral detachment [17, 18]. Though PAO can reorient the acetabulum to address the cause of the labral lesion and rotate the labrum away from the weight-bearing surface, the torn lesions can continue to give residual symptoms [17]. Ejnisman *et al*. discovered an association between increased acetabular anteversion and the rate of anterior labral tears found in patients with femoral acetabular impingement [18, 28]. Magnetic resonance imaging (MRI) studies comparing patients with acetabular retroversion to those without, identified a shorter superior labral length and less capsular thickening than in those with isolated acetabular dysplasia [21]. The increased load on the acetabular rim complex/labrum may result in a tension-type tear leading to labral detachment [18]. Within our review, Fuji *et al*. reported a high incidence (96%, 24 hips) of grade 1 or higher intra-articular labral lesions in Kellegren–Lawrence OA grade 1 hips [3], supporting that overloading of the hip leads to soft tissue pathology prior to osseous changes, and the argument that the presence of non-treated labral tears negatively influence PAO outcomes [3, 29, 30].

A variety of different outcome measures have been used in assessing the value of adjunct hip arthroscopy at the time of PAO including a variety of PROMS and return to sports. The PROMs used included: HOOS symptoms, HOOS ADL, HOS-SSS, WOMAC, VAS, iHOT-12/33, mHHS and NAHS (Tables III and IV). Riccardi reported that patient with symptomatic labral tears who underwent repair had lower HOOS-SSS post-operatively relative to patients who had PAO alone; however, these scores became equivalent by 1-year post-operatively [18]. In addition, patients who underwent arthroscopy for labral fixation in that study had greater improvements than those in the PAO alone cohort at the final follow-up [18]. The study by Gorozy *et al*. compared PROMs in patients who underwent PAO for hip dysplasia to patients who underwent PAO for hip dysplasia and concomitant arthroscopy for Cam deformity [19]. Their WOMAC scores for the PAO alone versus PAO with arthroscopy cohorts went from 74 ± 17 and 73 ± 19 to 91 ± 15 and 90.2, respectively [19]. The two groups EQ-5D scores went pre-operatively from 10 ± 2 in both groups to 8 ± 2 post-operatively in both study arms [19]. Ultimately, identifying non-significant result between the two groups, with further studies reporting no statistical difference between HOS-SS, NAHS or VAS scores at any point within their follow-up periods [2, 4, 7, 8, 12, 17, 18, 20]. Jimenez *et al*. studied the return to play in athletes who underwent PAO with arthroscopy and showed statistically significant (*P* < 0.001) post-operative improvements in mHHS (increase by 25.8 points), NAHS (increase by 28.6 points), HOS-SSS (increase by 35.8 point) and VAS (change of 3.2 points) [12]. Their group’s main finding was that 81.8% of their participants were able to return to the same level of sport or higher post-operation [12], which was greatly improved compared to patients who underwent an isolated PAO for symptomatic acetabular dysplasia whose returned-to-play at a rate of 67% [12, 31]. This statistical increase in return-to-play rate the authors contribute to arthroscopy treating intra-articular pathology at the time of PAO surgery, suggesting active patients could benefit from such intervention [12]. More recently, in 2023, Flemig *et al*. conducted the largest comparison study to-date of PAO’s with or without arthroscopy to directly assess if concurrent labral repair affected PROMs. Their study included 53 patients with confirmed labral tear on MRI who underwent a combined PAO with arthroscopic labral repair compared to a control group of 153 participants that underwent PAO alone [4]. They reported no significant difference between the two groups [4]. MRI evidence of labral detachment was present in 19% (33/170) of the PAO alone group, with 25 of these patients meeting the Minimal Clinical Importance Difference (MCID) for both reported PROMs (iHOT-33 and mHHS) [4]. Only a single patient required subsequent hip arthroscopy for labral repair for the ongoing hip pain after isolated PAO [4].

Adverse events were low across all studies, with accumulatively lateral femoral cutaneous nerve dysesthesia being reported as the most common complications occurring at a rate of 1.2% (13/1083). Superficial skin infections accounted for the second most common complication at 0.9% (10/1083), none of which required revision surgery. One study reported on the survivorship of combined hip arthroscopy with PAO to total hip replacement to be 97.4% at 8 years but going down to 54.6% based on the absence of radiographic progression of arthritis and 82% based on the achieved MCID on PROMs [8]. Cho *et al*. quote a 17% conversion rate of PAO to total hip replacement, though their own study only had one patient converting to a total hip replacement at 7.8 years out due to ongoing pain [8]. Berwin *et al*. reported two patients converting to a total hip replacement at 6 years and 13 years, respectively, and Matheney *et al*. had 17 out of 135 patients convert to a total hip replacement at an average of 6.1 years from PAO [9, 10]. Our reported conversion to total hip arthroplaty is lower than what is reported in the literature, which could be expalined by our average follow-up period being only 3.8 years. Within the literature, joint incongruity post-operatively has been identified as a predictor of PAO failures, and advanced intra-articular lesions associated with further progression of OA [3]. Subchondral bone exposure on the femoral head has been identified as an independent risk factor (*P* = 0.003) for progression of OA [3]. Sabbag *et al*. found statistical increase in Dindo–Clavien risk classification related to the age at which PAO surgery was performed, recognizing a 2.5 times higher risk of significant complications, grade III or IV, per decade of advanced age at the time of surgery [21]. They also suspected a three times increased risk of reoperation in patient populations who subsequently also were diagnosed with acetabular retroversion, however, those were not associated with PAO failures [21].

There are several limitations to our study. Our review includes studies that reported varying clinical outcomes measures, which precludes the ability to pool the study results using a meta-analysis technique. This is exacerbated by the large variations of PROMs included in our study and the fact that there is no agreed upon PROMs that is used universally within the PAO literature. More research is needed to develop a core outcome set that will improve the consistency and comparability of PROMs. Future studies can ensure that the most relevant and meaningful outcomes are consistently measured and reported by defining a standardized set of core outcomes. This will make data synthesis and meta-analysis easier, ultimately leading to the generation of more robust evidence to inform clinical practice decisions. Another limitation was the limited long-term follow-up of these studies, which our team believes does not express the conversion rates of PAO to THA. The average age at which a PAO was performed in our study was 28.4-year-old, with a mean follow-up of 3.7 (0.5–18) years. Further continued follow-up is required to determine the conversion rate to THA and POA longevity, as our accumulative years of follow-up was relatively low at 3.8 years. The scope of our paper focused on the arthroscopy component of the added procedure, and did not comment on, or analysis, femoroplasty or acetabuloplasty, which is a significant portion of the care of these patients. In addition, the use of only accepting English-derived studies is a limitation as one study was excluded during our full-text review as it was in another language, highlighting that the topic is being evaluated by investigators across the globe. From our study, we are unable to derive the add-on benefits of PAO compared to usual care because the majority of the included articles did not include control groups. The last major limitation from our study is the inherent relative bias due to the small number of articles, and subsequently cohorts, which met our inclusion criteria. Three groups of principle investigators are directly involved in 9 of our 14 selected articles, which allows for the possibility of potential overlap/duplication of results, though from our evaluation none of the included studies did so.

## CONCLUSION

There is a paucity of literature on clinical and patient-reported outcomes for patients with acetabular dysplasia undergoing concomitant PAO and hip arthroscopy. Patient-reported outcomes scores improve significantly after PAO with or without arthroscopy, with some evidence that active patients benefit from labral repair. Current studies have demonstrated that combined PAO with hip arthroscopy can provide excellent clinical outcome with low risk of complications. However, it still remains to be determined if combining hip arthroscopy to PAO provides a better outcome than PAO alone which requires a level I Randomized control trial study [32].

## Data Availability

The data underlying this article can be shared on request to the corresponding author.

## Level of evidence: IV

PROPERO Registration Number: CRD42022378731.
